# Health status and prognosis of COPD patients in relation to the FEV_1_/FVC ratio

**DOI:** 10.1038/s41533-025-00478-y

**Published:** 2026-01-08

**Authors:** Juan Wang, Björn Ställberg, Maria Hårdstedt, Kristina Bröms, Amir Farkhooy, Andreas Palm, Karin Lisspers, Marieann Högman, Andrei Malinovschi

**Affiliations:** 1 Department of Medical Sciences, Clinical Physiology, Uppsala University, Uppsala, Sweden; 2 Department of Medical Sciences, Respiratory-, Allergy- and Sleep Research, Uppsala University, Uppsala, Sweden; 3 Department of Public Health and Caring Sciences, General Practice, Uppsala University, Uppsala, Sweden; 4 Center for Clinical Research Dalarna-Uppsala University, Falun, Sweden; 5 Vansbro Primary Health Care Center, Vansbro, Sweden; 6 School of Medical Sciences, Örebro University, Örebro, Sweden

**Keywords:** Diseases, Health care, Medical research, Signs and symptoms

## Abstract

Whether COPD should be diagnosed using the lower limit of normal (LLN) or a fixed FEV_1_/FVC ratio <0.70 (FR) is debated. We compared symptom and disease burden in COPD patients with FEV₁/FVC below both thresholds (FR + /LLN + ) versus those between them (FR + /LLN-). This cohort study included 572 COPD patients from primary and secondary care in the central Swedish regions of Dalarna, Gävleborg, and Uppsala. FR + /LLN + COPD patients with FEV_1_ ≥ 60% predicted (n = 194) was compared to FR + /LLN- COPD patients (n = 85) in order to have similar FEV_1_ levels in both groups. The symptom burden was assessed using the modified British Medical Research Council scale of dyspnoea (mMRC), the COPD Assessment Test (CAT), and the Clinical COPD Questionnaire (CCQ). The disease burden was assessed by exacerbations and hospital admissions over the subsequent three years. The 279 studied patients (57% females) had a mean age of 68.2 years and a mean FEV_1_% predicted of 73.0%. The FR + /LLN+ group had comparable clinical characteristics to the FR + /LLN- group regarding FEV_1_% predicted (72.5 vs 74.2%), use of inhaled medicines (76.3 vs 76.5%), and previous exacerbations (23.2 vs 18.8%), all p-values > 0.05. Moreover, comparable prevalence of exacerbations and hospital admissions were found during the subsequent three years (31.7 vs 37.7%, and 4.8 vs 2.6%, respectively, all p-values > 0.05). Symptom burden was comparable for mMRC and CCQ, but the FR + /LLN- group had a higher CAT score than the FR + /LLN+ group (10.6 vs 12.6, p = 0.038), a finding also confirmed in adjusted analyses. FR + /LLN+ and FR + /LLN- COPD patients had relatively comparable symptom and disease burden, suggesting that not meeting the LLN criteria does not indicate a milder disease in clinically diagnosed COPD with comparable FEV_1_.

## Introduction

Chronic obstructive pulmonary disease (COPD) is a progressive lung condition characterized by airflow limitation that imposes a high burden on individuals and society through reduced quality of life, increased healthcare utilization, and substantial economic costs^1^.

There is a lack of general agreement on spirometric definition of chronic airflow limitation in currently available guidelines. The Global Initiative for Chronic Obstructive Lung Disease (GOLD) strategy document for COPD suggests using a fixed ratio (FR), a post-bronchodilator FEV_1_/FVC ratio <0.70, to define chronic airflow obstruction^2^. However, the FEV_1_/FVC ratio decreases with age and varies with sex^3^. Previous population studies have suggested that the use of FR may result in overdiagnosis of airflow obstruction in the elderly and underdiagnosis in young adults^4–6^. The proportion of individuals fulfilling the FR criteria, but not the lower limit of normal criteria (FR + /LLN-) was as high as 39% in a previous Swedish study^7^. One recent review concluded that the worldwide prevalence of COPD among adults aged 30–79 years was 10.3% according to the FR definition and 7.6% according to the LLN definition^8^. A post-bronchodilator FEV_1_/FVC ratio <LLN might be a more accurate criterion for COPD diagnosis^9–11^. ATS/ERS guidelines recommend therefore using the LLN, defined as the fifth percentile in healthy, never-smoking individuals, to define airflow obstruction^9^.

However, there is limited evidence regarding differences in health status and prognosis in patients with COPD diagnosed with FR compared to those fulfilling both the FR and LLN criteria. Compared to patients who met both criteria (FR + /LLN + ), those who met the FR criteria but not the LLN criteria (FR + /LLN-) had better health status with fewer symptoms^12–14^, less use of pulmonary medication^14^, less lung function impairment^7,12,13^, fewer hospitalizations for COPD exacerbations^15,16^, and reduced mortality^7,15^, but were more likely to report heart disease^13^. Moreover, FR + /LLN- COPD patients were often older and had higher BMI^12,13,17^. However, the FR + /LLN+ group in the above-mentioned studies includes patients with lower FEV_1_ than those in the FR + /LLN- group. Thus, it would be of interest to study specifically FR + /LLN+ patients with similar levels of FEV_1_ as FR + /LLN- patients.

The present cohort study included subjects from central Sweden with COPD diagnosed using the FR criteria. Our study has two aims. First, to compare various baseline clinical characteristics between the FR + /LLN+ and FR + /LLN- groups (both with similar FEV_1_ levels), including comorbidities, pulmonary medication, previous exacerbations, symptom burden, lung function, and inflammatory biomarkers. Second, to examine whether exacerbations and hospital admissions over the subsequent three years differed between the two groups.

## Materials and methods

### Study design and study population

**Fig. 1:**
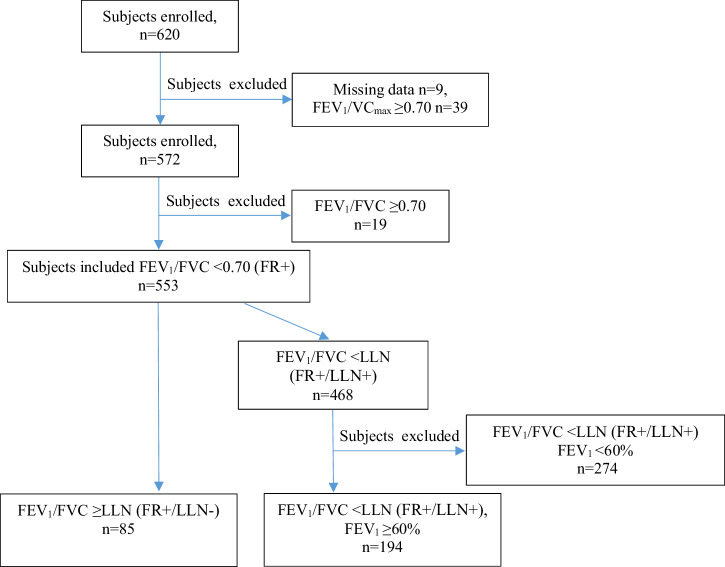
The flow chart of the study design. VC_max_ is the highest value of either FVC or SVC.

Subjects with a diagnosis of COPD (ICD code J44.0, J44.1, J44.8 and J44.9) from primary and secondary care settings from central Sweden regions of Dalarna, Gävleborg and Uppsala were invited to participate in the Tools Identifying Exacerbations in COPD study (TIE study)^18^. Postbronchodilatory spirometry was used to confirm COPD diagnosis. The criteria used for inclusion in the study was a ratio of forced expiratory volume in one second (FEV_1_) to the highest vital capacity value from a slow (slow vital capacity, SVC) or forced (forced vital capacity, FVC) maneuver <0.70. The inclusion study visit was performed from September 2014 to September 2016. A total of 620 subjects were initially included, of whom 48 were excluded, primarily due to not fulfilling the spirometry criteria when spirometry was performed at the study visit (Fig. 1)^18^. Among the included subjects (n = 572), 85.3% were from primary care settings and 14.7% were from secondary care settings. A questionnaire was used to obtain information on demographics, education, current smoking habits, comorbidities, exacerbations, pulmonary medication and symptom burden at inclusion. Measurement of lung function and collection of blood samples were performed during the study visit at inclusion. The study visit was delayed by four weeks in case of an ongoing exacerbation occurred. The current analyses included all subjects with a ratio of FEV_1_/FVC < 0.70 obtained at inclusion; 553 out of 572 subjects fulfilled this criterion. Of these 553 patients, 194 met both the FR and LLN criteria (FR + /LLN + ) and had FEV_1_ ≥ 60%, and 85 met only the FR criterion (FR + /LLN-). The criterion of FEV_1_ ≥ 60% was selected and used in order to have similar FEV_1_ levels in the two groups in the present analyses. The group fulfilled FR + /LLN+ and FEV_1_ < 60% (n = 274) was not studied in the present analyses.

### Lung function

Post-bronchodilator spirometry was performed 15 min after administration of 400 μg salbutamol, in accordance with the ATS/ERS standardization^19^, using a SpiroPerfect Spirometer (Welch Allyn, Skaneateles Falls, NY, USA) or a Jaeger MasterScreen PFT (Erich Jaeger GmbH, Würzburg, Germany). Data on forced expiratory volume in one second (FEV_1_), slow vital capacity (SVC) and forced vital capacity (FVC) were measured. The predicted values of FEV_1_ and FVC were calculated using the GLI 2012 equations^9^. The ratio FEV_1_/FVC was calculated using measured values.

The LLN was defined as a z-score for FEV_1_/FVC below the fifth percentile according to the GLI 2012 lung function reference equations^9^. The LLN was used to classify subjects into the FR + /LLN+ (concordant) and FR + /LLN- (discordant) groups in our study.

### Questionnaire

Data on demographic characteristics (sex, age, and education level) and current smoking status (smokers/non-smokers) were obtained from the inclusion questionnaire. Data on height and weight were measured during the clinical visit at inclusion.

Information on comorbidities (asthma, chronic bronchitis, ischemic heart disease, heart failure, atrial fibrillation, hypertension, stroke, diabetes, cancer, osteoporosis, sleep apnea, rheumatic disease, and anxiety/depression) was obtained from the inclusion questionnaire. Data on the use of any form of inhaled corticosteroids (ICS), use of long-acting β₂-adrenergic agonist (LABA), and use of long-acting muscarinic antagonist (LAMA) were also collected.

Symptom burden was assessed through questionnaire at inclusion. Three symptom scales were used: the modified British Medical Research Council (mMRC) scale of dyspnea, which grades breathlessness from 0–4^20,21^, the COPD Assessment Test (CAT), an 8-item measure of the impact of COPD on health status^22^, and the COPD Clinical Questionnaire (CCQ), a 10-item tool of assessing symptoms, functional state, and mental state^23^.

### Exacerbations of COPD

An exacerbation was defined as an acute healthcare visit and/or a course of oral corticosteroids and/or antibiotics prompted by COPD deterioration. Data on exacerbation history during the year preceding inclusion, and subsequent exacerbations and COPD-related hospital admissions over the following three years were retrieved from medical records.

### Biomarkers for inflammation

Collected blood samples were used to analyse inflammation biomarkers including blood neutrophils (B-Neu), blood eosinophils (B-Eos), C-reactive protein (CRP), and plasma fibrinogen, using clinical routine equipment as previously described^24^. Data on blood neutrophils (B-Neu) and blood eosinophils (B-Eos) were expressed as 10^9^/L. Data on CRP and plasma fibrinogen were expressed as mg/L. Exhaled nitric oxide (with an exhalation flow of 50 mL/s, Fe_NO50_) was measured according to current recommendations^25^.

### Statistics

The program STATA 18 was used for the statistical analysis. Qualitative variables were expressed as absolute frequencies and percentages, while quantitative variables were summarized as means and standard deviations (SD) for normally distributed data, or medians and interquartile ranges (IQR) otherwise. Proportions of categorical variables were compared between groups using the Chi-square test or Fisher’s exact test, while differences in continuous variables were assessed using the t-test or Mann-Whitney U test. Multiple comparisons were adjusted using the Benjamini–Hochberg procedure^26^. Associations between different factors (smoking, education, comorbidities, symptom burden, and inflammatory biomarker) and the binary outcome of discordant/ concordant were analysed using logistic regression models (each variable was modelled separately), adjusting for sex, age, and BMI. Associations were expressed as odds ratios (OR) with 95% confidence intervals (CI). Kaplan–Meier analyses were conducted to estimate the probabilities of events between groups over time with respect to time to first exacerbation and time to first hospital admission after inclusion.

### Ethics

The study protocols were approved by the Regional Review Board in Uppsala, Sweden (Dnr 2013/358) on 25 September 2013. All subjects gave written informed consent.

## Results

In total, 57.0% of the subjects were female, and 30.1% were current smokers. The FR + /LLN+ group had a higher proportion of female patients and smokers, was slightly younger, and had a lower BMI (Table 1).

**Table 1:** The demographics, treatment, exacerbations, symptom burden, and spirometry of the participants at inclusion (n = 279).

	Total n = 279	Discordant FR + /LLN- ^a^ n = 85	Concordant FR + /LLN+ ^b^ FEV_1_ ≥ 60% n = 194	p	Adjusted p ^e^
Female sex (n %) ^c^	159 (57.0%)	31 (36.5%)	128 (66.0%)	**0.000005**	**0.00007**
Age (mean ± SD) ^d^	68.2 ± 7.8	71.0 ± 6.5	66.9 ± 8.1	**0.0001**	**0.0007**
BMI (mean ± SD) ^d^	27.2 ± 4.6	28.6 ± 5.0	26.6 ± 4.3	**0.0009**	**0.004**
Current smoker (n, %) ^c^	84 (30.1%)	17 (20.0%)	67 (34.5%)	**0.015**	**0.042**
Education: at least three years in high school (n, %) ^c^	58 (20.9%)	13 (15.5%)	45 (23.3%)	0.140	0.218
Any use of ICS (n, %) ^c^	156 (55.9%)	46 (54.1%)	110 (56.7%)	0.689	0.803
Any use of LAMA or LABA (n, %) ^c^	148 (53.1%)	45 (52.9%)	103 (53.1%)	0.981	0.981
Any use of ICS, LAMA or LABA (n, %) ^c^	213 (76.3%)	65 (76.5%)	148 (76.3%)	0.974	0.981
Any exacerbation one year before inclusion (n, %) ^c^	61 (21.9%)	16 (18.8%)	45 (23.2%)	0.416	0.530
mMRC ≥2 (n, %) ^c^	90 (32.3%)	32 (37.7%)	58 (29.9%)	0.202	0.283
CAT (mean ± SD) ^d^	11.2 ± 7.2	12.6 ± 7.1	10.6 ± 7.2	**0.038**	0.089
CCQ (mean ± SD) ^d^	1.41 ± 1.00	1.58 ± 1.02	1.33 ± 0.98	0.052	0.104
FEV_1_% predicted (mean ± SD) ^d^	73.0 ± 11.0	74.2 ± 14.2	72.5 ± 9.2		
FVC % predicted (mean ± SD) ^d^	94.8 ± 16.6	85.6 ± 16.7	98.8 ± 14.9		
FEV_1_/FVC (%) (mean ± SD) ^d^	59.9 ± 6.5	66.1 ± 2.4	57.2 ± 5.9		

Bold values indicate p < 0.05.

^a^FR + /LLN- means FEV_1_/FVC < 0.70 & FEV_1_/FVC ≥ LLN.

^b^FR + /LLN+ means FEV_1_/FVC < 0.70 & FEV_1_/FVC < LLN.

^c^Chi-square test.

^d^t-test.

^e^p values were adjusted using Benjamini–Hochberg procedure.

**Fig. 2:**
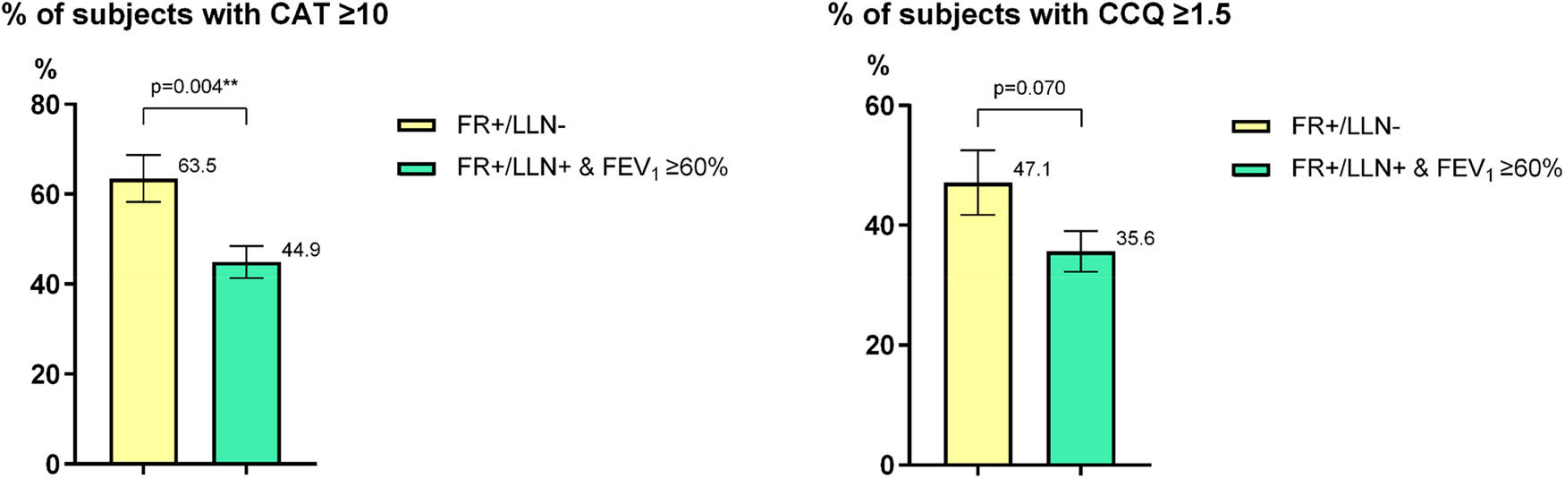
The proportion of participants with CAT ≥ 10 and CCQ ≥ 1.5 at inclusion. FR + /LLN- means FEV_1_/FVC < 0.70 & FEV_1_/FVC ≥ LLN. FR + /LLN+ means FEV_1_/FVC < 0.70 & FEV_1_/FVC < LLN.

The clinical characteristics of the included subjects are summarized in Table 1. The FR + /LLN+ group generally had similar clinical characteristics to the FR + /LLN- group in terms of any form of pulmonary medications, exacerbations during the past year, mMRC and CCQ score, and FEV_1_% predicted. However, the FR + /LLN+ group had a higher FVC % predicted, and a lower FEV_1_/FVC ratio. Moreover, a lower proportion of subjects in the FR + /LLN+ group had a CAT score ≥10. The proportions of subjects with a CCQ score ≥1.5 were comparable in both groups (Fig. 2).

The most common comorbidities were hypertension, chronic bronchitis, asthma, and anxiety/depression (Table 2). The prevalence of comorbidities was similar between the two groups, except that atrial fibrillation and hypertension were less common in the FR + /LLN+ group. The comparison of inflammatory biomarkers between the two groups showed no significant difference in the levels of B-Neu, B-Eos, Fe_NO50_, CRP, and fibrinogen (Table S1).

**Table 2:** The prevalence of comorbidities of the participants at inclusion (n = 279).

Comorbidities	Total n = 279	Discordant FR + /LLN- ^a^ n = 85	Concordant FR + /LLN+ ^b^ & FEV_1_ ≥ 60% n = 194	p ^c^	Adjusted p ^d^
	n %	n %	n %		
Asthma	80 (28.9%)	21 (24.7%)	59 (30.7%)	0.308	0.435
Chronic bronchitis	83 (29.9%)	26 (30.6%)	57 (29.5%)	0.859	0.859
Ischemic heart disease	27 (9.7%)	12 (14.1%)	15 (7.7%)	0.097	0.316
Heart failure	14 (5.0%)	6 (7.1%)	8 (4.1%)	0.301	0.435
Atrial fibrillation	32 (11.5%)	18 (21.2%)	14 (7.3%)	**0.001**	**0.013**
Hypertension	127 (45.5%)	49 (57.7%)	78 (40.2%)	**0.007**	**0.046**
Stroke	24 (8.6%)	11 (12.9%)	13 (6.7%)	0.090	0.316
Diabetes	20 (7.2%)	9 (10.6%)	11 (5.7%)	0.143	0.320
Cancer	38 (13.4%)	14 (16.5%)	24 (12.4%)	0.367	0.435
Osteoporosis	25 (9.0%)	11 (12.9%)	14 (7.2%)	0.123	0.320
Sleep apnea	34 (12.2%)	13 (15.3%)	21 (10.8%)	0.294	0.435
Rheumatic disease	37 (13.3%)	10 (11.8%)	27 (13.9%)	0.626	0.678
Anxiety/depression	63 (22.7%)	16 (18.8%)	47 (24.5%)	0.300	0.435

Bold values indicate p < 0.05.

^a^FR + /LLN- means FEV_1_/FVC < 0.70 & FEV_1_/FVC ≥ LLN.

^b^FR + /LLN+ means FEV_1_/FVC < 0.70 & FEV_1_/FVC < LLN.

^c^Chi-square test.

^d^p values were adjusted using Benjamini–Hochberg procedure.

Logistic regression analyses, adjusted for sex, age, and BMI, revealed that only osteoporosis and higher CAT scores were independently associated with lower odds of being classified as concordant (FR + /LLN + ). All other characteristics showed no significant differences in odds between the groups (Table 3). Additional analyses showed that the association with osteoporosis was significant only in models that included sex as a covariate.

**Table 3:** Associations between smoking, education, comorbidities, symptom burden, inflammation biomarker, and being as concordant among participants from inclusion (n = 279).

		Discordant FR + /LLN- ^a^ n = 85	Concordant FR + /LLN+ ^b^ & FEV_1_ ≥ 60% n = 194	p
			OR (95%CI) ^c^	
Current smoker	Yes	1.00	0.99 (0.49,2.00)	0.980
Education	At least three years in high school	1.00	1.72 (0.82,3.62)	0.152
Comorbidities	Ischemic heart disease	1.00	0.76 (0.31,1.86)	0.553
	Atrial fibrillation	1.00	0.63 (0.28,1.45)	0.280
	Hypertension	1.00	0.86 (0.48,1.53)	0.598
	Stroke	1.00	0.61 (0.24,1.57)	0.308
	Diabetes	1.00	0.70 (0.26,1.90)	0.487
	Osteoporosis	1.00	0.33 (0.13,0.85)	**0.021**
CAT	Continuous ^d^	1.00	0.96 (0.92,0.996)	**0.029**
	≥10	1.00	0.46 (0.26,0.83)	**0.009**
CCQ	Continuous ^e^	1.00	0.79 (0.60,1.05)	0.100
	≥1.5 (vs <1.5)	1.00	0.69 (0.39,1.24)	0.214
Inflammation biomarker	B-Neu (10^9^/L) ^f^	1.00	1.11 (0.90,1.36)	0.326

Bold values indicate p < 0.05. All variables with a p value < 0.2 from Table 1, Table 2 and Table S1 are included in this table.

^a^FR + /LLN- means FEV_1_/FVC < 0.70 & FEV_1_/FVC ≥ LLN.

^b^FR + /LLN+ means FEV_1_/FVC < 0.70 & FEV_1_/FVC < LLN.

^c^Logistic regression models, adjusting for sex, age and BMI.

^d^Per 1 unit increase of CAT score was applied in the model.

^e^Per 1 unit increase of CCQ score was applied in the model.

^f^Per 1 unit (10^9^/L) increase of B-Neu was applied in the model.

Sensitivity analyses after exclusion of subjects with a history of asthma revealed similar results, except that there were no significant differences on education level, having atrial fibrillation and hypertension (Table S2 and S3). In additional logistic regression analyses excluding individuals with asthma, no significant difference in osteoporosis was observed between groups (Table S4).

**Fig. 3:**
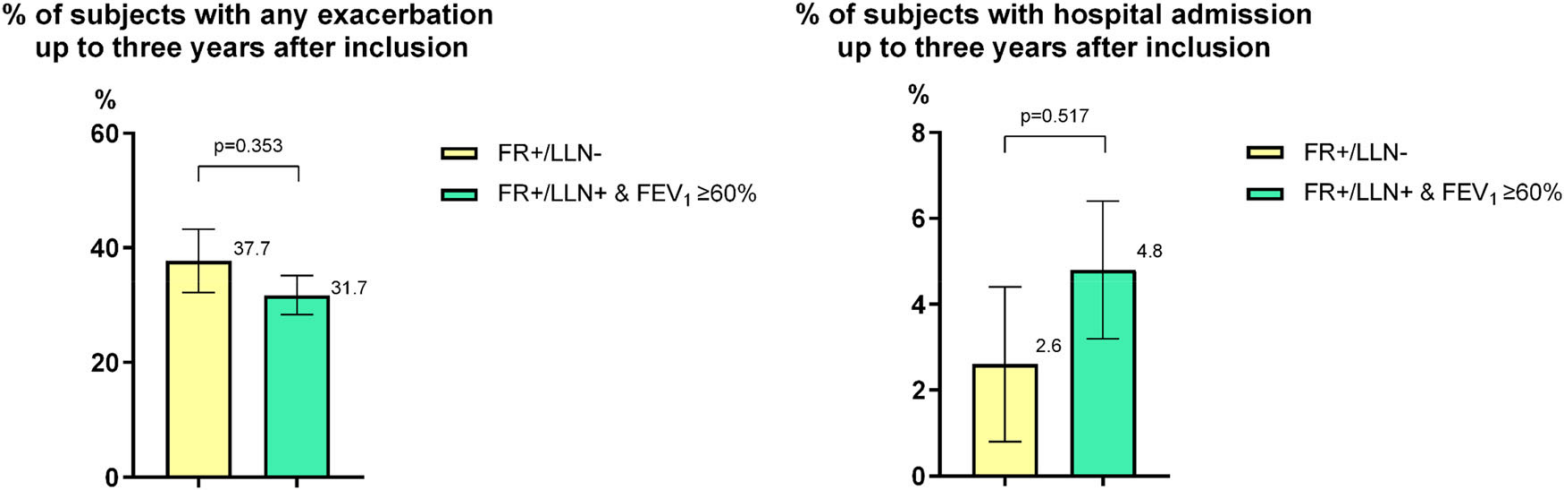
The proportion of participants with any exacerbation and any hospital admission up to three years after inclusion among those were still alive three years after inclusion. FR + /LLN- means FEV_1_/FVC < 0.70 & FEV_1_/FVC ≥ LLN. FR + /LLN+ means FEV_1_/FVC < 0.70 & FEV_1_/FVC < LLN.

**Fig. 4:**
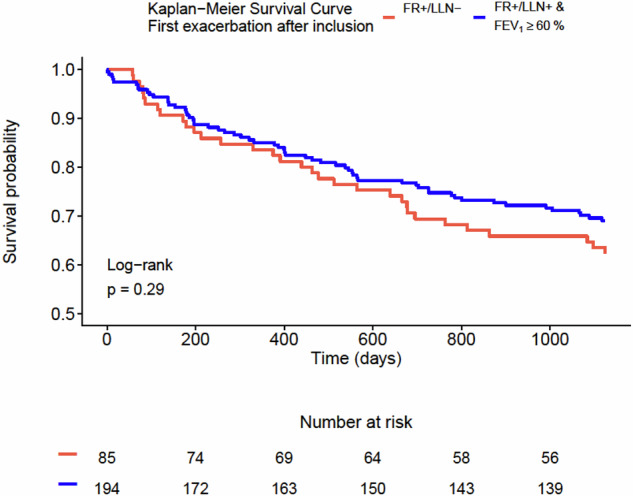
Kaplan–Meier graph showing the two groups in relation to time until first exacerbation after inclusion. FR + /LLN- means FEV_1_/FVC < 0.70 & FEV_1_/FVC ≥ LLN. FR + /LLN+ means FEV_1_/FVC < 0.70 & FEV_1_/FVC < LLN.

**Fig. 5:**
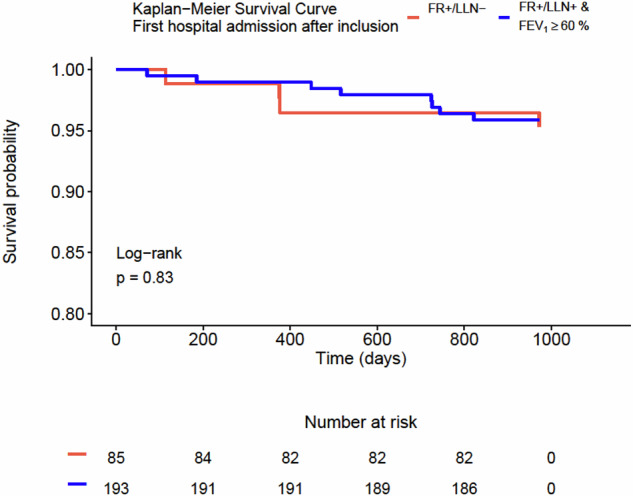
Kaplan–Meier graph showing the two groups in relation to time until first hospital admission after inclusion. FR + /LLN- means FEV_1_/FVC < 0.70 & FEV_1_/FVC ≥ LLN. FR + /LLN+ means FEV_1_/FVC < 0.70 & FEV_1_/FVC < LLN.

Among subjects who were still alive three years after inclusion (n = 263), 33.5% of subjects experienced at least one exacerbation over the following three years after inclusion, and 4.2% were hospitalized due to an exacerbation during the same period. However, no significant difference was found between the two groups in the proportion of subjects experiencing any exacerbation or hospital admission up to three years after inclusion (Fig. 3). Additional Kaplan-Meier analyses with regard to time to first exacerbation and time to first hospital admission after inclusion showed no significant differences between the two groups (Figs. 4 and 5).

## Discussion

In this study, the comparison of subjects with FR + /LLN+ (FEV₁ ≥60%) and FR + /LLN- demonstrated comparable clinical characteristics. We observed no differences in inhaled medication, exacerbations in the year prior to inclusion, the occurrence of any exacerbation or hospital admission due to an exacerbation during the subsequent three years, mMRC and CCQ scores, or reported comorbidities. However, subjects meeting both FR and LLN criteria with an FEV_1_ ≥ 60% were more likely to be women, younger, and to have lower BMI. This group also had lower CAT scores and a lower prevalence of osteoporosis after controlling for sex, age, and BMI. Finally, no differences were observed between the two groups in the investigated levels of inflammatory biomarkers.

In our study, the FR + /LLN+ and FR + /LLN- groups demonstrated comparable use of inhaled medication, exacerbations, and hospital admissions over the subsequent three years. This contrasts with findings from a Spanish patient cohort, which reported a higher prevalence of COPD-related hospitalisations among concordant (FR + /LLN + ) patients compared to their discordant (FR + /LLN-) counterparts^15^. Similarly, a large-scale study from the UK identified greater risks of both moderate-to-severe and severe exacerbations in individuals meeting both diagnostic criteria (FR + /LLN + ) during a three-year follow-up period^16^. The most plausible explanation is that these studies included subjects with poorer lung function (e.g., lower FEV_1_), as no selection based on FEV_1_ levels was made in the above-mentioned studies. In contrast, we had comparable FEV_1_ levels in the two groups and were not able to find any differences in the risk of exacerbations and hospitalisations between FR + /LLN+ and FR + /LLN-individuals.

Our finding regarding the difference in sex is consistent with previous research showing that the fixed ratio criterion tends to under-diagnose airflow obstruction in younger females^27,28^. There is a sex difference regarding the LLN levels in women and men, with higher levels in women^9^. The LLN in women might be closer to 0.70, leading to fewer women in the discordant group in our study. Nevertheless, previous studies comparing COPD patients who met both diagnostic criteria versus those meeting only the FR criterion reported no significant sex differences^7,12–17^. The FR + /LLN+ subjects in our study were younger than the FR + /LLN- subjects, which is consistent with existing literature showing that the fixed ratio criterion tends to over-diagnose airflow obstruction in older adults^13,27,28^. One population-based study in Sweden suggested that 0.70 is a relatively good proxy for LLN in individuals aged 50–64^29^, with discordance increasing after 65 years of age^3^. Our findings on age differences also align with previous studies^12,13,17^, although those studies did not restrict inclusion to individuals with FEV_1_ ≥ 60% of predicted. The reason for the lower BMI observed in the FR + /LLN+ group compared to the FR + /LLN- group is unclear, but our finding is consistent with previous studies showing that the FR + /LLN+ group tends to have a lower BMI^12,13,17^.

Our study showed that subjects in the FR + /LLN+ group had lower CAT scores (in logistic regression models). However, this contrasts with findings from a UK study, which reported that individuals meeting both the FR and LLN criteria (FR + /LLN + ) had higher CAT scores and exhibited more respiratory symptoms, including cough, sputum production, and wheeze^13^. Similarly, a Swedish population-based study found that individuals meeting both criteria (FR + /LLN + ) had a higher prevalence of long-standing cough and dyspnoea on exertion^14^. We believe these differences in results are due to our selection of individuals with well-preserved FEV_1_, in order to match the FEV_1_ levels in the FR + /LLN group.

In the logistic regression analyses, osteoporosis was negatively associated with the FR + /LLN+ group, while no significant differences were found for other comorbidities. There are well-established connections between COPD and osteoporosis, including the higher prevalence of osteoporosis in COPD patients, as well as the influence of COPD severity and the role of inhaled corticosteroid use in increasing the risk of osteoporosis^30,31^. A review and meta-analyses indicated that osteoporosis was associated with a significant reduction in pulmonary function (reduced FEV_1_/FVC ratio and FEV_1_) among patients with COPD^32^. The FR + /LLN+ group was younger and had lower BMI that might explain a higher likelihood of having better bone health. Finally, the fact that the significant association with osteoporosis was found only after adjustment for sex likely reflects that some of the difference was masked in Table 2 due to fewer women in the FR + /LLN- group.

Subjects in the FR + /LLN+ and FR + /LLN- groups exhibited comparable levels of B-Neu, B-Eos, Fe_NO50_, CRP, and fibrinogen in the present study. To our knowledge, no previous studies have specifically compared these biomarkers between individuals meeting only the FR criterion and those fulfilling both the FR and LLN criteria.

### Strengths and limitations

There are several strengths in our study. A large number of symptomatic subjects recruited from primary care who met the FR criteria were included in our analyses. Objective measurements of lung function (post-bronchodilator spirometry) and inflammatory biomarkers were performed at inclusion. Moreover, a broad range of data was collected, including demographics, smoking habits, comorbidities, exacerbations, and symptom burden (using mMRC, CAT, and CCQ scores). Our study has some limitations. We did not invite patients with COPD randomly from the three regions, limiting somewhat the generalizability of our findings. Despite a relatively large population, the FR + /LLN- group was relatively small. This limited sample size may have reduced the power to detect differences in symptom and disease burden between the two groups. The absence of 2:1 matching or propensity score matching in the present study is a limitation. Information on comorbidities was obtained through a self-reported questionnaire. Another limitation is that, although the FR + /LLN+ (FEV_1_ ≥ 60%) group appeared to have a lower risk of osteoporosis, the small number of cases limited the strength of this conclusion. Additionally, the study setting involved patients already diagnosed with COPD, which may make some inferences regarding the potential use of these criteria for diagnosis less robust.

## Conclusion

The present study found that most of the clinical characteristics, including ICS, LABA or LAMA treatment, exacerbation history, hospital admissions, comorbidities, and inflammatory biomarkers, were comparable between the FR + /LLN+ (FEV_1_ ≥ 60%) and FR + /LLN- groups. These findings suggest that COPD patients, with similar FEV_1_ values, may experience comparable symptoms and disease burden, irrespective of whether their FEV_1_/FVC ratio is decreased according to LLN.

## Supplementary information

Supplementary Information

## Data Availability

Data cannot be made freely available as they are subject to secrecy in accordance with the Swedish Public Access to Information and Secrecy Act, but can be made available to researchers upon request, after approval from the Swedish Ethical Review Authority has been obtained.
