# Supplementary material for: Health status and prognosis of COPD patients in relation to the FEV1/FVC ratio
Source: NPJ Prim Care Respir Med. 2026 Jan 8;36:4. doi: 10.1038/s41533-025-00478-y (PMC12789526; doi:10.1038/s41533-025-00478-y)
Supplement: Supplementary file 1 — Supplementary Information [file 41533_2025_478_MOESM1_ESM.pdf]

Supplement

Table S1. Inflammatory biomarkers of the participants at inclusion (n=279).

Inflammation biomarkers	**Total** n=279	**Discordant** FR+/LLN- [a] n=85	**Concordant** FR+/LLN+ [b] & FEV_1 ≥60% n=194	p [c]
	Median (IQR)	Median (IQR)	Median (IQR)	
B-Neu (10^9/L)	4.3 (3.6,5.4)	4.1 (3.4,5.1)	4.4 (3.6,5.4)	0.117
B-Eos (10^9/L)	0.2 (0.1,0.3)	0.2 (0.1,0.3)	0.2 (0.1,0.3)	0.865
FE_{NO50} (ppb) [d]	13.5 (9.0,20.3)	13.6 (10.2,22.0)	13.5 (8.5,20.3)	0.654
CRP (mg/L)	2.3 (1.1,4.2)	2.0 (1.0,4.4)	2.3 (1.1,4.1)	0.755
Fibrinogen (mg/L) [e]	3.4 (2.9,3.9)	3.4 (2.9,3.9)	3.3 (2.9,3.9)	0.374

The number of missing data points for inflammation biomarkers is not reported if there were fewer than 5 cases of missing data.

[a] FR+/LLN- means FEV_1/FVC <0.70 & FEV_1/FVC ≥LLN.

[b] FR+/LLN+ means FEV_1/FVC <0.70 & FEV_1/FVC <LLN.

[c] Mann-Whitney U test.

[d] Missing data for FE_{NO} n=14.

[e] Missing data for fibrinogen n=14.

Table S2. The demographics, treatments, exacerbations, symptom burden, and spirometry of the participants at inclusion, excluding those with asthma (n=197).

	**Total** n=197	**Discordant** FR+/LLN- [a] n=64	**Concordant** FR+/LLN+ [b] & FEV_1 ≥60% n=133	p	Adjusted p [e]
Female sex (n %) [c]	106 (53.8%)	22 (34.4%)	84 (63.2%)	**0.00015**	**0.0018**
Age (mean ± SD) [d]	68.5 ± 7.6	71.3 ± 6.2	67.2 ± 7.6	**0.0003**	**0.0018**
BMI (mean ± SD) [d]	27.0 ± 4.4	28.0 ± 4.7	26.65 ± 4.1	**0.027**	**0.081**
Current smoker (n, %) [c]	64 (32.5%)	14 (21.9%)	50 (37.6%)	**0.027**	**0.081**
Education: at least three years in high school (n, %) [c]	42 (21.4%)	8 (12.5%)	34 (25.8%)	**0.034**	0.082
Any use of ICS (n, %) [c]	89 (45.2%)	29 (45.3%)	60 (45.1%)	0.979	0.979
Any use of LAMA or LABA (n, %) [c]	101 (51.3%)	33 (51.6%)	68 (51.1%)	0.954	0.979
Any use of ICS, LAMA or LABA (n, %) [c]	140 (71.1%)	46 (71.9%)	94 (70.1%)	0.862	0.979
Any exacerbation one year before inclusion (n, %) [c]	38 (19.3%)	12 (18.8%)	26 (19.6%)	0.894	0.979
mMRC ≥2 (n, %) [c]	58 (29.4%)	22 (34.4%)	36 (27.1%)	0.292	0.438
CAT (mean ± SD) [d]	10.9 ± 7.2	11.9 ± 6.9	10.5 ± 7.3	0.191	0.377
CCQ (mean ± SD) [d]	1.39 ± 1.00	1.51 ± 0.97	1.33 ± 0.95	0.220	0.377
FEV_1 % predicted (mean ± SD) [d]	73.3 ± 11.4	74.9 ± 14.8	72.6 ± 9.3		
FVC % predicted (mean ± SD) [d]	94.9 ± 17.4	86.4 ± 17.4	99.0 ± 15.9		
FEV_1/FVC (%) (mean ± SD) [d]	60.1 ± 6.4	66.0 ± 2.3	57.2 ± 5.9		

Bold values indicate p<0.05.

[a] FR+/LLN- means FEV_1/FVC <0.70 & FEV_1/FVC ≥LLN.

[b] FR+/LLN+ means FEV_1/FVC <0.70 & FEV_1/FVC <LLN.

[c] Chi-square test.

[d] t-test.

[e] p values were adjusted using Benjamini–Hochberg procedure.

Table S3. The prevalence of comorbidities of the participants at inclusion, excluding those with asthma (n=197).

Comorbidities	**Total** n=197	**Discordant** FR+/LLN- [a] n=64	**Concordant** FR+/LLN+ [b] & FEV_1 ≥60% n=133	p	Adjusted p [e]
	n %	n %	n %		
Chronic bronchitis [c]	54 (27.4%)	19 (29.7%)	35 (26.3%)	0.619	0.624
Ischemic heart disease [c]	21 (10.7%)	8 (12.5%)	13 (9.8%)	0.624	0.624
Heart failure [d]	8 (4.1%)	4 (6.3%)	4 (3.0%)	0.238	0.476
Atrial fibrillation [c]	22 (11.2%)	12 (18.8%)	10 (7.6%)	**0.020**	0.204
Hypertension [c]	88 (44.7%)	33 (51.6%)	55 (41.4%)	0.177	0.425
Stroke [c]	20 (10.2%)	10 (15.6%)	10 (7.6%)	0.081	0.315
Diabetes [c]	12 (6.1%)	5 (7.8%)	7 (5.3%)	0.484	0.581
Cancer [c]	23 (11.7%)	12 (18.8%)	11 (8.3%)	0.034	0.204
Osteoporosis [c]	17 (8.6%)	7 (10.9%)	10 (7.5%)	0.424	0.565
Sleep apnea [c]	28 (14.2%)	11 (17.2%)	17 (12.8%)	0.407	0.565
Rheumatic disease [c]	28 (14.2%)	7 (10.9%)	21 (15.8%)	0.361	0.565
Anxiety/depression [c]	44 (22.6%)	10 (15.6%)	34 (26.0%)	0.105	0.315

Bold values indicate $p<0.05$.

[a] FR+/LLN- means FEV_1/FVC <0.70 & FEV_1/FVC ≥LLN.

[b] FR+/LLN+ means FEV_1/FVC <0.70 & FEV_1/FVC <LLN.

[c] Chi-square test.

[d] Fisher's exact test.

[e] p values were adjusted using Benjamini–Hochberg procedure.

Table S4. Associations between smoking, education, comorbidities, symptom burden, inflammation biomarker, and being as concordant among participants from inclusion, excluding those with asthma (n=197).

		**Discordant** FR+/LLN- [a] n=85	**Concordant** FR+/LLN+ [b] & FEV_1 ≥60% n=194	p
			OR (95%CI) [c]	
Current smoker	Yes	1.00	1.19 (0.55,2.56)	0.654
Education	At least three years in high school	1.00	2.31 (0.93,5.74)	0.072
Comorbidities	Ischemic heart disease	1.00	1.03 (0.37,2.86)	0.961
	Atrial fibrillation	1.00	0.70 (0.26,1.87)	0.477
	Hypertension	1.00	1.16 (0.58,2.29)	0.680
	Stroke	1.00	0.59 (0.22,1.63)	0.313
	Diabetes	1.00	0.86 (0.24,3.06)	0.817
	Osteoporosis	1.00	0.44 (0.14,1.38)	0.158
CAT	Continuous [d]	1.00	0.96 (0.91,1.004)	0.072
	≥10	1.00	0.49 (0.25,0.96)	**0.037**
CCQ	Continuous [e]	1.00	0.79 (0.56,1.11)	0.177
	≥1.5 (vs <1.5)	1.00	0.74 (0.38,1.45)	0.379
Inflammation biomarker	B-Neu (10^9/L) [f]	1.00	1.08 (0.85,1.37)	0.544

Bold values indicate p<0.05. All variables with a p value <0.2 from Table 1, 2 and Table S1 are included in this table.

[a] FR+/LLN- means FEV_1/FVC <0.70 & FEV_1/FVC ≥LLN.

[b] FR+/LLN+ means FEV_1/FVC <0.70 & FEV_1/FVC <LLN.

[c] Logistic regression models, adjusting for sex, age and BMI.

[d] Per 1 unit increase of CAT score was applied in the model.

[e] Per 1 unit increase of CCQ score was applied in the model.

[f] Per 1 unit (10^9/L) increase of B-Neu was applied in the model.
